# Health literacy in an Israeli elderly population

**DOI:** 10.1186/s13584-019-0328-2

**Published:** 2019-07-10

**Authors:** Michal Hochhauser, Michael Brusovansky, Maria Sirotin, Katerina Bronfman

**Affiliations:** 1 Department of Occupational Therapy, Faculty of Health Sciences, Ariel University, Ramat Hagolan 65, 4070000 Ariel, Israel; 2 Beilinson Hospital, Hospital in Petah Tikva, Petah Tikva, Israel; 3 Leumit Health Services, Tel Aviv-Yafo, Israel; 4 Loewenstein Hospital, Rehabilitation center in Ra’anana, Ra’anana, Israel

**Keywords:** Comprehension, Education status, Elderly, Health literacy, Medical information

## Abstract

**Background:**

Health literacy is important for patients’ comprehension of the health and medical messages conveyed to them and their meaning for them so that they can better manage their health. The aim of the study was to examine the level of health literacy within the elderly population. The hypothesis was that health literacy would be inadequate, and related to demographic variables.

**Method:**

Sixty men and women over the age of 65 who volunteered to participate in the study completed a 13-item health literacy questionnaire.

**Results:**

Overall, the level of health literacy among the participants was mostly inadequate. They reported difficulty in reading medical material in Hebrew and understanding the doctor, thus requiring assistance (20%); difficulty in reading medical documents, completing medical forms and understanding medical terms; difficulty in reading the leaflet attached to medications (33%), test results (40%) and medical information written in English (66%) and difficulty searching the internet for information (53.3%)**.** The level of health literacy was associated with education while the best profile for adequate health literacy was for those who spoke Hebrew and completed secondary education.

**Conclusions:**

Medical teams have an obligation to be alert and attentive to the level of health literacy of elderly patients and to modify communication and information to an accommodating degree, so that elderly patients can better manage their health**.**

**Electronic supplementary material:**

The online version of this article (10.1186/s13584-019-0328-2) contains supplementary material, which is available to authorized users.

## Introduction

Health literacy is defined as ‘the combination of personal competencies and situational resources needed for people to access, understand, appraise and use information and services to make decisions about health. It includes the capacity to communicate, assert and act upon these decisions.’ (Broder et al., 2018) [1]. In this way, health literacy is presented as a set of *individual capacities* that allow the person to acquire and use new information. These capacities may decline with aging or pathologic processes that impair cognitive function. Health literacy is also defined as ‘the competencies and actions required to make judgements and decisions in everyday life concerning health include health care, disease prevention, and health promotion’ (Sørensen et al., 2012) [2]. This concept asserts that patients must be able to comprehend the health messages being conveyed to them and their meaning for them so that they can have better control over their health (Baker, 2006) [3]. Such comprehension is even more crucial nowadays, since today’s patients are partners in their own treatment and have the autonomy to make informed decisions about the most suitable treatment for themselves (Nutbeam. 2008; Mehudar, 2014;). [4, 5]

Several health literacy reviews (Peerson and Saunders, 2009; Sørensen et al., 2012; Mehudar, 2014) [2, 5, 6] identified various health literacy dimensions; some are narrow and restrictive while others are broad and expansive. A more restrictive dimension defines health literacy as the ability of a person to use reading and writing skills to convey and receive messages. A broader dimension defines health literacy as the ability of an individual to obtain, process, comprehend and assimilate medical knowledge in order to reach appropriate medical decisions and understand the treatment instructions (Nutbeam, 2001) [7]. The accepted approach which stems from the aforementioned literature relates to three dimensions of health literacy. The first, functional health literacy, relates to the possession of literacy and sufficient knowledge to acquire and act on information on defined health risks and recommended health services use. The second, communicative health literacy, relates to the skills required from an individual to manage his health in partnership with professionals. The third and highest level is critical health literacy, expressed as the ability of an individual to critically analyze information, raise his own awareness of his health problems, and take action that will enable him to achieve informed medical decisions with the aim of preventing or reducing health risks and to improve his quality of life (Sørensen et al., 2012) [2]. This classification of health literacy helps to distinguish between the different skills that progressively enable greater autonomy in decision making, in addition to a wider range of health actions that extend from personal behaviors to social action that address the underlying determinants of health, thus increasing individual and community empowerment (Broder et al., 2018, 1]. In recent years two additional types of health literacy were defined. The first, digital health literacy, is expressed by the ability to seek, find, understand and evaluate information from the internet, and implement the obtained information in order to treat a health problem and find a solution for it (Breinin and Netter, 2009) [8]. The second, media health literacy, is expressed by the ability to identify health-related content appearing in the media, whether revealed or hidden, understand its impact on health behavior, critically evaluate the presented content, and to indicate an intention to act and/or respond to the exposure to such content (Levin–Zamir et al., 2011). [9]. All these dimensions assume that a population with a sufficient level of health literacy is adept at forming appropriate decisions and improve its state of health (Levin–Zamir et al., 2016) [10]. The concept of health literacy has also developed in two distinctive contexts; one in which strengthening citizens’ competencies are the focus and the other where the community/organizations decrease the complexity of the health care system, to better guide, facilitate and empower citizens to sustainably manage their health (Sorenson et al. 2015, [11]).

Surveys conducted in different countries around the world show that in developed countries, and more so in developing countries, a large proportion of the population has limited health literacy (Sørensen et al., 2013; Mehudar 2014) [5, 12]. A large proportion of people around the world, even when they are able to understand most of the health information, still find it difficult to understand and analyze medical information that is written in complex, sophisticated language. Limitations to health literacy lead to a situation where individuals are often unable to be active partners in their treatment plan or to independently manage their diseases efficiently and effectively.

Limited health literacy has been shown to be linked to negative health outcomes. The first negative outcome is low accessibility to health communication and medical information. Due to heavily burdened healthcare systems, it is difficult to convey the vast and complex information required for patients to better manage their health, such that in order to better manage their medical treatment or exercise their healthcare rights, patients are required to obtain medication and health information independently. Thus, patients with limited health literacy find it challenging to manage their health (Weiss, 2007; Breinin and Netter, 2009) [8, 13]. The second negative outcome is a lack of information about individually adapted, available and accessible health and medical services, leading to a lack of use of such services, particularly those dealing with primary prevention (Weiss, 2007; Kanj and Mitic, 2009) [13, 14]. A third negative outcome is higher illness and mortality rates among populations with limited health literacy due to a lack of knowledge about treatment options, the importance of responding to treatment, and the most effective way to use medications (Peerson and Saunders, 2009; Berkman et al., 2011) [6, 15]. A fourth negative outcome is an economic burden. Health expenses among patients with inadequate health literacy are much higher than those of patients with intermediate or adequate health literacy. The reasons for this are mainly due to more frequent visits to the family doctor; longer hospitalizations due to medical complications arising from not following treatment instructions properly; more visits to hospital emergency wards due to complications arising from a lack of communication about how to effectively manage common, non-complex health situations; and a deteriorating state of health due to errors in taking medications (Levin-Zamir et al., 2012; Mehudar, 2014) [5, 16]. A fifth negative outcome is that those with inadequate health literacy are more likely to report difficulties with instrumental activities of daily living and activities of daily living, specifically limitations in normal work activities due to physical health and pain (Wolf et al., 2006) [17].

Studies have found that the level of health literacy is related, among other things, to socio-demographic variables, such as age, gender and education (Pelikan et al., 2012; Sørensen et al., 2012; Sørensen et al., 2013) [2, 12, 18]. Limited health literacy is common mainly among the elderly population (above 65) and within this population has been found to be more common among women than among men, and among the poorly-educated than the highly-educated (Tiller et al., 2015, 19]. Such limited health literacy has an impact on this population, which is characterized by a multitude of chronic illnesses that persist for many years and lead to increased and prolonged need of health services.

According to a report based on the Israeli Central Bureau of Statistics (CBS) (Brodsky, Shnoor, & Be’er [19], 22% of the Jewish elderly were born in Israel, compared to 73% of the total population. Out of the 78% elderly immigrants, 23% immigrated to Israel in the last 26 years (since 1990), the vast majority from the former Soviet Union. The data indicates that Hebrew is not the mother tongue for almost two thirds of the elderly and quite a large portion are most likely not fluent in the Hebrew language. Data collected in Israel in 2006 by the UNESCO Institute for Statistics indicated that although 97.1% of the population can read and write, a relatively large proportion of people demonstrate limited health literacy. There are two reasons that can explain this limitation: first, the language in which health and medical information is written, at times is highly sophisticated and complex making it difficult to understand, and second, as in many countries with immigrants, Hebrew is not the mother tongue for a relatively large proportion of the population (Levin et al., 2012; Levin–Zamir 2016) [10, 16]. It is worth noting that the Arab elderly population in Israel share some of the same characteristics, at the same time having additional distinct traits. Future studies in the Arab population are warranted.

The elderly population is characterized, more than younger age groups, in traditional patterns of life. This is reflected in a significant gap in the level of education between men and women. Over the years, however, older women have raised their level of education at higher rates than older men. For example, in the years 1995–2015, the percentage of elderly women with16 years of education and over grew by 9.2% (from 6 to 19%), compared to the increase of 2.2% among older men (from 12 to 27%). As a result, education gaps between men and women have narrowed, and in general 22% of the elderly have tertiary education. Another interesting statistic is that the percentage of elderly living alone in Israel stands at 23%. In the rest of the developed countries, this percentage is higher (for example, 35% or more in the Scandinavian countries). The percentage of those living alone increases with age (31% among those aged 75+, compared with 17% among those aged 65–74). Women live alone more than men (32 and 12%, respectively). Another interesting fact is that elderly people use the computer at a lower rate than the general population; 49% compared to 72% of those aged 20+. The proportion of elderly who use the internet also stands at 49%, with 65% of them using their mobile phone. The most common uses of the internet are to search for information (94%), send emails (80%) and participate in social networks (66%). Despite the relatively low rate of use of technology in the elderly, over the past 13 years (2002–2015), the rate of elderly people’s use of the internet increased by 2.8% (from 6% at the beginning of the period to 49% at the end), whereas increase among the general population was 4.2 (from 32 to 77%). Overall, over the years the level of education of men and women aged 65+ has increased, and the gaps between the elderly and the younger age groups in education and use of internet have diminished, yet the gaps still exist. Despite these gaps, to the knowledge of the authors, a study on health literacy in the elderly in Israel has not recently been published. As technology evolves the complexity of attaining medical information has become more challenging. Adding to that are the unique demographic features in the growing elderly population (i.e. 84% not Israeli born) particularly in the geographical area in which the study was conducted. Therefore, the aim of this study is to examine the level of health literacy within this population. The research hypothesis claims that the level of health literacy in the elderly population will be inadequate and will be characterized by a need to receive assistance from others to read or understand medical material; a low ability to comprehend medical explanations; and a low ability to seek and obtain medical information. The study also assumes that the level of health literacy in these three areas will be linked to the following demographic variables: gender, education, state of health, ability to read in Hebrew and English and type of residence.

## Methods

The participants were recruited from the geographical area of the university where 2120 elderly citizens are listed. The population in this includes a large number of immigrants (82%) in an otherwise non -heterogeneous community. A large portion immigrated from the past Soviet Union, some from Arab speaking countries and a smaller portion from the United States. We had sought out an evidence-based measure of effect size yielded from a published study that is conceptually similar to the current study (Tiller 2015, 19] to calculate the sample size needed for a chi-square analysis. We used the reported effect size that provides the absolute difference between proportion of people in the two groups of interest; low and high education that had the categorical outcome of adequate or inadequate level of health literacy. The software G*Power calculation showed that 38 participants were required for a sample representing the population at an acceptable significance level of 0.5 at the probability of 80%.

Sixty men and women aged 65 and over (M ± SD = 74.13 ± 7.33) participated in the study, selected by convenience sampling, where 36.7% were men, and 63% were women, 8.3% of them had primary education, 31.7% had secondary education and 60% had tertiary education, 75% of them live with a partner, children or caregiver and 25% live alone. After receiving ethical approval from the Ethics Committee Board of Ariel University, the researchers approached the directors of two senior citizen recreational centers in the area and received confirmation to conduct the study. There were no exclusion criteria as the population attending the recreational centers have mental capacities enabling verbal exchange in an interview. The interviews were conducted in Hebrew. Trilingual interviewers and an on-site translator addressed language barriers. A variety of means were used to recruit participants such as bulletin board notices, word -of-mouth and through the center’s administrative and professional staff (social worker, volunteers). Sixty participants responded positively to whom the researchers explained the aims of the research and guaranteed anonymity and confidentiality of the obtained information. Surveys were conducted individually for 15 min on average.

The survey comprised a 13-item questionnaire based on the short European Health Literacy questionnaire (HLS-EU-Q16) research instrument adapted for the general population in Israel to the elderly population (Additional file 1). The tool was reduced from 16 items to 13 items. Following a pilot study, the researchers felt that the combination of age and language barrier require a greater length of time for the interview and that the participants became fatigued. In addition, other aspects were brought up such as measuring level of assistance (not only competencies but practicalities), thus we adjusted the items (e.g. combined 2 in one category). The 13 items examined the subjects’ level of health literacy from three perspectives. First, we examined the degree to which the subject requires assistance to read and understand medical information (items 1, 2, 5, 6, 9). For example: “I need assistance to understand the doctor’s words”. Cronbach’s alpha test revealed a correlation of 0.67 among these five items. Second, the degree to which the subject reads and understands medical information related to his medical condition (items 3, 4, 7, 8, 13). For example: “I understand the medical terms related to my medical condition”. Cronbach’s alpha test revealed a correlation of 0.64 among these five items. Third, the degree to which the subject is able to obtain medical information, obtain health information from the internet, compare sources of information and make an informed medical decision (items 10, 11, 12). For example: “I know how to obtain all of the information I need to understand my state of health”. Cronbach’s alpha test revealed a correlation of 0.74 among these three items. All 13 items were examined on a 5-point Likert scale, where 1 = “Strongly agree” and 5 = “Strongly disagree”. In addition, the questionnaire included two items that examined the subject’s ability to read in Hebrew and in English, as well as six demographic items: age, gender, education, state of health, type of residence and language (mother tongue).

### Statistical analysis

After descriptive statistics were tabulated and frequencies were examined, associations between demographic variables and items reflecting health literacy were tested using the Chi-square design. After observing that there were specific associations in a number of demographics, a 2 way MANOVA was conducted to analyze these specific demographics. Analyses was performed by SPSS (version 23) with statistical significance set at *p* ≤ 0.05.

## Results

The results of the health literacy analysis are presented according to their reported frequency on the three levels: inadequate, marginal and adequate (Table 1). To this end, the scales were combined such that the first two factors “Strongly Agree” and “Agree” reflect an inadequate level of health literacy, the level “Neither agree or disagree” reflects a marginal level of health literacy, and the levels “Disagree” or “Strongly disagree” reflect an adequate level of health literacy. In factors 3 and 4 the scales were reversed. The means and standard deviations are presented according to the Likert scale (1–5), such that a cutoff for adequate/ inadequate levels of literacy were determined by an average of 2.5.

**Table 1:** Frequencies of three levels of health literacy

*Health Literacy items*	Mean ± SD	Inadequate	Marginal	Adequate
*Reading and understanding medical material*				
I need assistance to read the hospital release letter or summary of doctor’s appointment	3.45 ± 1.48	30%	15%	55%
I need assistance to understand the doctor’s words	3.85 ± 1.27	18.3%	18.3%	63.4%
I need assistance to complete medical forms	3.35 ± 1.58	35%	6.7%	58.3%
I feel confident completing medical forms ^a^	2.85 ± 1.62	41.7%	8.3%	50%
I need assistance from others to understand my test results	3.02 ± 1.43	40%	18.3%	41.7%
*Reading medical information in Hebrew and in English*				
I have difficulty reading medical information about my state of health written in English	2.33 ± 1.50	63.3%	13.3%	23.4%
I have difficulty reading medical information about my state of health written in Hebrew	3.58 ± 1.58	23.3%	16.7%	60%
*Understanding medical information about the subject’s health*				
I understand everything the doctor says during my appointment	2.25 ± 1.28	21.7%	11.7%	66..7%
I understand all the instructions printed on the leaflet attached to my medication	2.55 + 1.48	28.3%	10%	61.7%
I understand the medical terms related to my state of health	2.92 ± 1.28	30%	28.3%	41.7%
*Seek and obtain information and compare sources of information*				
I know how to obtain all the information I require and to understand my state of health	2.83 ± 1.33	35%	18.3%	46.7%
I know how to seek information about my state of health on the internet	3.42 ± 1.54	53.3%	8.3%	38.4%
I am accustomed to comparing the information I obtain from different sources in order to reach an informed medical decision that is the most appropriate for me	3.52 ± 1.51	56.7%	13.3%	30%

^a^Scale was reversed to calculate frequencies

The first finding relates to the degree of assistance required by the subject to read and understand medical material independently (items 1, 2, 5, 6, 9). Between one fifth and one third of the subjects reported an inadequate level of health literacy. The second finding relates to the degree to which the subject has difficulty reading medical information about their state of health, written in Hebrew or English (items 3, 4). Conversely, a much lower, but still notable, percentage (23.3%) of the subjects reported inadequate health literacy as reflected in the difficulty they experience reading medical information about their state of health in Hebrew. The third finding relates to the degree to which subjects understand medical information about their state of health (items 7, 8, 13). Here too, a marked percentage of between one fifth and one third of the subjects reported inadequate health literacy. The fourth finding relates to the degree to which the subject is able to seek and obtain information and compare sources of information in order to make an informed decision (items 10, 11, 12). Yet again, a marked percentage of one third to one half of the subjects reported inadequate health literacy.

This study also examined the relationships between demographic variables and different aspects of health literacy (Table 2).

**Table 2:** Results of Chi-square test and descriptive statistics for health literacy by level of education, language and English comprehension

Items	Level of Education	Health Literacy			χ^2^*(df)*
		Adequate	Marginal	Inadequate	
Degree of assistance required to read and understand medical material					
*Item 1.* Assistance to read hospital release letter/ summary of doctor’s appointment	Low	0 (0%)	2 (40%)	3 (60%)	6.91 (2)*
	High	33 (60%)	7 (12.7%)	15 (27.3%)	
*Item 2*. Assistance to understand doctor’s words	Low	1 (20%)	1 (20%)	3 (60%)	6.8 (2)*
	High	37 (67.3%)	10 (18.2%)	8 (14.5%)	
*Item 5.* Assistance to complete medical forms	Low	0 (0%)	0 (0%)	5 (100%)	10.13 (2)**
	High	35 (63.6)	4 (7.3)	16 (29.1)	
*Item 6.* Feels confident completing medical forms	Low	0 (0%)	0 (0%)	5 (100%)	7.64 (2)*
	High	30 (54.5)	5 (9.1)	20 (36.4)	
*Item 9.* Assistance in understanding test results	Low	0 (0%)	0 (0%)	5 (100%)	8.18 (2)*
	High	25 (45.5)	11 (20%)	19 (34.5%)	
Degree of difficulty reading medical information written in Hebrew or English about state of health					
*Item 4.* Difficulty to read medical information in Hebrew	Low	1 (20%)	4 (80%)	0 (0%)	15.9***
	High	35 (63.6%)	6 (10.9%)	14 (25.5%)	
Degree to which subjects understand medical information about their state of health					
*Item 7.* Understands everything the doctor says during an appointment	Low	1 (20%)	3 (60%)	1 (20%)	12.71 (2)**
	High	39 (70.9%)	4 (7.3%)	12 (21.8%)	
*Item 13.* Understands the medical terms related to state of health	Low	1 (20%)	0 (0%)	4 (80%)	6.7 (2)*
	High	24 (43.6%)	17 (30.9%)	14 (25.5%)	
Ability to seek/obtain/ compare information	Language				
*Item 10.* Knows how to obtain all the information needed to understand state of health	Hebrew	9 (45%)	7 (25%)	4 (30%)	9.7 (4)*
	OtheRussian Russian	4 (28.6%)	2 (14.3%)	8 (57.1%)	
	Russian	15 (57.7%)	2 (7.7%)	9 (34.6%)	
	Understands English				
*Item 11.* Knows how to seek information about state of health on the internet	Good ^a^	12 (60%)	3 (15%)	5 (25%)	9.8 (2)**
	Poor	11 (27.5%)	2 (5%)	27 (67.5%)	

^a^Good and Very Good were unified

*p ≤ .05, **p ≤ .01, ***p ≤ .001

A chi-square test of association revealed no significant relationships for the demographic variables; age, gender and living arrangements, however demographics of education yielded associations with adequate health literacy in most items as well as language and English comprehension with several items. Health status (not reported in the table) was significantly associated with needing assistance to understand the doctor’s words, *X*^2^ (2, *N* = 60) = 7.9, *p* = .02.

**Fig. 1:**
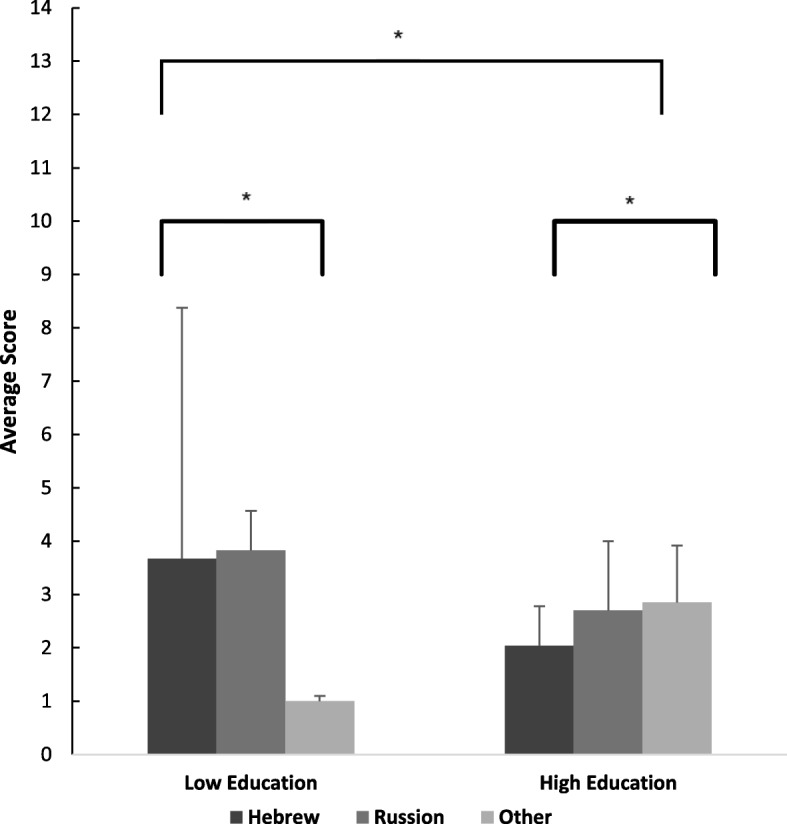
Differences in health literacy factor “understanding medical information” between Hebrew, Russian and foreign language speakers with high/low levels of education. Lower averages indicate better (adequate) health literacy

Following the series of Pearson correlations, a two-way multivariate analysis of variance (MANOVA) was conducted to test the hypothesis that there would be one or more mean differences between educational levels (low and high) and language as reflected in the health literacy item scores (Fig. 1). Low scores indicate greater (adequate) health literacy.

A statistically significant main effect of education on health literacy was obtained, *F* (4, 51) = 4.65, *p* < .003; Pillai’s trace V = 0.27, partial η^2^ = .27, but the main effect of language on health literacy was not significant, *F* (8, 104) = 4.65, *p* = .45; Pillai’s trace V = 0.14. The high-education group showed significantly greater (adequate) health literacy than the low-education group, requiring less assistance to read and understand medical material, *F* (1, 5.31) = 11.92, *p* = .001), and greater ability to seek and obtain information and compare sources of information in order to make an informed decision, *F* (1, 5.6) = 5.25, *p* = .03), while there were no significant differences between the groups in the items reflecting the ability to read or understand medical information about one’s state of health.

There was a significant interaction between the level of education and language in the domain “understanding medical information”, *F* (1,36) = 10.125, *p* = .003. Multiple comparisons showed that highly educated Hebrew speakers were significantly more health literate (in the factor “understanding medical information”) than foreign language speakers (other than Russian) (*p* = .04) and significantly more health literate than Russian speakers (*p* = .05), but there were no differences between Russian speakers and foreign language speakers (*p* = .69). Upon inspecting low education, results revealed that foreign language speakers (other than Russian) were significantly more health literate than Hebrew speakers (*p* = .05), and more than Russian speakers (*p* = .036), but there were no differences between Hebrew speakers and Russian speakers (*p* = .89). These results indicate that understanding medical information was greater for those who had higher education and spoke Hebrew.

## Discussion

The current study extends on conclusions from prior studies in that there is an interplay between education and language: one backs the other and can serve as a coping strategy and help promote health literacy. At the same time, they do not constitute a substitute for inadequate health literacies that do not exist among native Israelis with the same educational level. The findings of this study provide evidence that the level of health literacy among the elderly in the studied population is somewhat inadequate, and mainly related to education and language. Indeed, one fifth to one third of the elderly require assistance from others to read or understand medical material, have difficulty understanding medical explanations, and have difficulty seeking and obtaining medical information. These findings are consistent with those of the European Health Literacy Survey (HLS-EU) (Pelikan et al., 2012) [18] conducted in 2012 and with an Israeli study performed in 2008 (Breinin and Netter, 2009) [8]. However, they are inconsistent with the Israel Health Literacy Survey (Levin-Zamir, 2016) [10] in which health literacy was not seen to be significantly dependent on age. This may be attributed to the fact that the survey included the general population whereas the current study focused on the elderly population thus accentuating language, education and digital limitations which often characterize the elderly in Israel.

In general, participants with higher levels of education reported more adequate health literacy. This finding is in line with the CARLA study which analyzed the elderly East Germany population (Tiller et al., 2015) [20], and additional studies which point out the association between limited health literacy and low education (Benson et al.2002; Chew et al., 2004; Baker et al., 2007) [21–23]. Interestingly, a different study conducted in Germany (Vogt et al., 2018) [24] did not find that education was related to health literacy, which may be explained by cultural aspects. Specifically, we found that those with higher education are less likely to require medical assistance (i.e. doctors’ summaries, test results), similarly to a study which noted that education and health-related literacy are associated in recalling standard instructions (Chin et al., 2017) [25]. An interesting finding of this study was that a stronger ability to seek information, obtain information and compare sources of information on the internet was directly linked to a higher level of education, regardless of language, meaning that education provides the individual with strategic compensations for language barriers. The study sample data revealed that 47% of the participants feel that they perform moderately to adequately in obtaining information on the internet, alike the data provided by the Israeli CBS where 49% of the general elderly reported internet fluency. This is highly encouraging as a strategy for obtaining medical information. Nonetheless, highly educated Hebrew speakers compared to highly educated Russian speakers or foreign language speakers, specifically have better abilities in understanding medical information given by the doctor during an appointment, the instructions printed on the leaflet attached to medications, and medical terms related to state of health, thus having a better understanding regarding their state of health. Yet, highly educated Russian speakers have better abilities in understanding medical information than other foreign language speakers. This in part, may be explained by the possibility that a large percentage of the medical staff in the location of the research are Russian speaking. The advantages of education and speaking the native language with respect to health literacy are supported in additional studies (Gazmararian et al., 1999) [26]. An additional finding was that English comprehension was related to adequate health literacy. This may be explained by the fact that most evidence based health literature found on the internet is written in English. Similarly, in the low educated population, foreign language speakers had more adequate health literacy possibly explained by English speakers using compensatory strategies. These findings are consistent with the research literature (Sørensen, 2013; Pelikan et al., 2016) [12, 18]. It can be taken into account that this convenience sample is overly weighted towards persons with extensive prior formal education and that the more educated tended to report better health literacy than the less educated. Nonetheless, health literacy was still deficient with more than 1/3 of the respondents having difficulty with each category of comprehension. As the convenience sample has an over-representation of well- educated elderly, it is likely that for the general elderly population, the prevalence of health literacy limitations is even greater than the 1/3 rate found for the convenience sample.

An additional finding relates to the living arrangements of the study sample in which those living alone constitutes 22% of the elderly population alike the statistics provided by the CBS of those living alone among the Israeli elderly. While correlations were not found between living arrangements and health literacy, living alone could indicate too little social support to buffer the negative consequences of low health literacy (Lee et al., 2004) [27].

We found a single association between low health status and inadequate health literacy in the factor of requiring assistance to understand the doctor. This can be attributed to the fact that in cases where individuals with inadequate health literacy do not receive assistance, their health status worsens. Multiple studies reported that individuals with inadequate health literacy had worse health status than those with adequate health literacy (Miller, 2004; Protheroe et al., 2017) [28, 29]. Moreover, those with lower health literacy were less likely to engage in preventive healthcare (Scott et al., 2002) [30] thus causing deterioration in their state of health which may place an economic burden on the healthcare system (Weiss, 2007; Kanj et al., 2009; Peerson et al., 2009; Berkman et al., 2011; Shalom and Farber, 2012; Levin et al., 2012) [6, 13–16, 31].

We did not find associations between health literacy and living arrangements nor with age. This is in line with a study which demonstrated that age was not a significant factor for health literacy (Buchbinder et al., 2006) [32]. Nevertheless, the literature shows conflicting results regarding age (Cutilli, 2007) [33] but we can conclude that among the elderly, a significant proportion of patients are characterized by inadequate health literacy. In light of the results, it is important to define and create strategies to promote health literacy in the elderly which in turn can positively impact health status, health empowerment, and longevity. More recently the IUHPE Global Working Group on Health Literacy has defined four health literacy action areas to advance health literacy and health promotion policy: policy, intervention, measurement and research, and building capacity (Broder et al., 2018, 1].

## Conclusions

Overall, the elderly have been identified as most vulnerable to inadequate health literacy. In the current study, the level of health literacy reported was mostly inadequate; comprehension of medical material (e.g. test results, forms, leaflets) and doctors’ instructions; completing medical forms; difficulty searching the internet for information, thus requiring constant assistance. The level of health literacy was associated with education while the best profile for adequate health literacy was for those who spoke Hebrew and completed secondary and tertiary education.

We must note the limitations of the study. It was conducted on a relatively small sample of elderly adults who volunteered to participate in the study. As there were no specific inclusion criteria, a positive health literacy bias might be created as the result of volunteer participants who take initiative and have more awareness. On the other hand, the fact that they have inadequate health only enhances the problem. An additional limitation was that where 60% of the study sample had tertiary education, only 22% of the elderly in the general Israeli population have tertiary education. This only widens the gap of health literacy in the general elderly population between those who are educated and non- educated. Thus, policy makers and health care managers should address the problems arising from health literacy deficits among the elderly.

Additionally, several demographics which may have enhanced the interpretation of the results were not tested, such as rural versus urban living. Further studies should analyze additional demographics included in larger study sizes.

Last, the adjusted items in the questionnaire yielded intermediate internal consistency and should be reevaluated for reliability in additional populations.

## Implications

The implications of our findings suggest that all medical staff be alert and attentive to elderly patients’ levels of health literacy and convey information in ways they can understand in order to capably manage their health. There is a need to enhance identification methods for those at risk of inadequate health literacy, for example, the role of education and language. This may also imply the need to include routine assessment of health literacy in assessment procedures for older adults. We should be aware that the use of electronic medical resources among older adults may present obstacles in attaining important health information, therefore this population may require longer appointments which often place a burden on the medical system. In addition, we should search for intervention strategies on mitigating the negative effects of low health literacy in this group and examine their effectiveness on health outcomes and impact on healthcare cost.

## Additional file

Additional file 1: Health literacy questionnaire for the geriatric population. (DOCX 19 kb)

## Data Availability

Please contact author for data requests.
