# Supplementary material for: Health literacy in an Israeli elderly population
Source: Isr J Health Policy Res. 2019 Jul 10;8:61. doi: 10.1186/s13584-019-0328-2 (PMC6621944; doi:10.1186/s13584-019-0328-2)
Supplement: Supplementary file 1 — Health literacy questionnaire for the geriatric population. (DOCX 19 kb) [file 13584_2019_328_MOESM1_ESM.docx]

Additional file 1

Research questionnaire

Please rank your level of agreement with the following statements

|  | Very true of me | True of me | Somewhat true of me | Untrue of me | Very untrue of me |
| --- | --- | --- | --- | --- | --- |
| 1. I need assistance to read the hospital release letter or summary of the doctor’s appointment | 1 | 2 | 3 | 4 | 5 |
| 1. I need assistance to understand the doctor’s words | 1 | 2 | 3 | 4 | 5 |
| 1. I find it difficult to read medical information in English about my condition | 1 | 2 | 3 | 4 | 5 |
| 1. I find it difficult to read medical information in Hebrew about my condition | 1 | 2 | 3 | 4 | 5 |
| 1. I need assistance to complete medical forms | 1 | 2 | 3 | 4 | 5 |
| 1. I feel confident completing medical forms | 1 | 2 | 3 | 4 | 5 |
| 1. I understand everything the doctor tells me during an appointment | 1 | 2 | 3 | 4 | 5 |
| 1. I understand all the instructions printed on the leaflet attached to my medications | 1 | 2 | 3 | 4 | 5 |
| 1. I need others to assist me in understanding my test results | 1 | 2 | 3 | 4 | 5 |
| 1. I know how to obtain all the information I need to understand my state of health | 1 | 2 | 3 | 4 | 5 |
| 1. I know how to seek information about my state of health on the internet | 1 | 2 | 3 | 4 | 5 |
| 1. I am used to comparing information that I obtain from different sources in order to reach the most suitable medical decision for myself | 1 | 2 | 3 | 4 | 5 |
| 1. I understand the medical terms related to my state of health | 1 | 2 | 3 | 4 | 5 |

1. How would you define your ability to read in Hebrew?

1. Very good 2. Good 3. Fairly good 4. Not so good 5. Not good at all

15. How would you define your ability to read in English?

1. Very good 2. Good 3. Fairly good 4. Not so good 5. Not good at all

16. Age________

17. Gender: 1. Male 2. Female

18. Education: 1. Below secondary 2. Secondary 3. Tertiary

19. State of health: 1. Very good 2. Good 3. Not good 4. Bad

20. Mother tongue:

21. Do you live: 1. Alone 2. With a family member
